# Characterization of *VHL* missense mutations in sporadic clear cell renal cell carcinoma: hotspots, affected binding domains, functional impact on pVHL and therapeutic relevance

**DOI:** 10.1186/s12885-016-2688-0

**Published:** 2016-08-17

**Authors:** Caroline Razafinjatovo, Svenja Bihr, Axel Mischo, Ursula Vogl, Manuela Schmidinger, Holger Moch, Peter Schraml

**Affiliations:** 1 Institute of Surgical Pathology, University Hospital Zurich, Zurich, Switzerland; 2 Oncology Clinic, University Hospital Zurich, Zurich, Switzerland; 3 Department of Medicine, St. Joseph Hospital Vienna, Vienna, Austria; 4 Department of Medicine I, Clinical Division of Oncology and Comprehensive Cancer Center, Medical University of Vienna, Vienna, Austria

**Keywords:** Clear cell renal cell carcinoma, VHL, Missense mutations, Binding domains, pVHL stability, Therapy

## Abstract

**Background:**

The VHL protein (pVHL) is a multiadaptor protein that interacts with more than 30 different binding partners involved in many oncogenic processes. About 70 % of clear cell renal cell carcinoma (ccRCC) have *VHL* mutations with varying impact on pVHL function. Loss of pVHL function leads to the accumulation of Hypoxia Inducible Factor (HIF), which is targeted by current targeted treatments. In contrast to nonsense and frameshift mutations that highly likely nullify pVHL multipurpose functions, missense mutations may rather specifically influence the binding capability of pVHL to its partners. The affected pathways may offer predictive clues to therapy and response to treatment. In this study we focused on the *VHL* missense mutation pattern in ccRCC, and studied their potential effects on pVHL protein stability and binding partners and discussed treatment options.

**Methods:**

We sequenced *VHL* in 360 sporadic ccRCC FFPE samples and compared observed and expected frequency of missense mutations in 32 different binding domains. The prediction of the impact of those mutations on protein stability and function was assessed *in silico*. The response to HIF-related, anti-angiogenic treatment of 30 patients with known *VHL* mutation status was also investigated.

**Results:**

We identified 254 *VHL* mutations (68.3 % of the cases) including 89 missense mutations (35 %). Codons Ser65, Asn78, Ser80, Trp117 and Leu184 represented hotspots and missense mutations in Trp117 and Leu 184 were predicted to highly destabilize pVHL. About 40 % of *VHL* missense mutations were predicted to cause severe protein malfunction. The pVHL binding domains for HIF1AN, BCL2L11, HIF1/2α, RPB1, PRKCZ, aPKC-λ/ι, EEF1A1, CCT-ζ-2, and Cullin2 were preferentially affected. These binding partners are mainly acting in transcriptional regulation, apoptosis and ubiquitin ligation. There was no correlation between *VHL* mutation status and response to treatment.

**Conclusions:**

*VHL* missense mutations may exert mild, moderate or strong impact on pVHL stability. Besides the HIF binding domain, other pVHL binding sites seem to be non-randomly altered by missense mutations. In contrast to LOF mutations that affect all the different pathways normally controlled by pVHL, missense mutations may be rather appropriate for designing tailor-made treatment strategies for ccRCC.

**Electronic supplementary material:**

The online version of this article (doi:10.1186/s12885-016-2688-0) contains supplementary material, which is available to authorized users.

## Background

Renal cell carcinoma (RCC) is the ninth most common cancer type worldwide [1–3]. There are three main RCC subtypes that are determined by their histologic features: papillary RCC, chromophobe RCC and clear cell RCC (ccRCC), the latter is known to be closely related with mutation of the Von Hippel-Lindau gene (*VHL*). ccRCC represents 75 % of all RCC cases and is also the most aggressive form of this cancer type [4].

Standard treatment for localized disease is surgery (partial or total nephrectomy), and targeted therapy as well as novel immunotherapies for metastasizing tumors. Despite all the recent efforts, the optimization of efficient therapies remains a major challenge for most cases of metastatic RCC [1, 5].

The *VHL* gene is a tumor suppressor gene of 639 coding nucleotides distributed over three exons and located at chromosome 3p25.3 [6]. The *VHL* gene product (pVHL) has been identified as a multiadaptor protein, interacting with more than 30 different binding partners [7]. Its best described function is to target other proteins for ubiquitination and proteasomal degradation as component of an E3 ubiquitin protein ligase, also termed VBC-cul2 complex [8, 9]. Among its targets are the hypoxia-inducible factor (HIF) subunits 1α and 2α (HIF1α and HIF2α), which upregulate many genes, such as VEGF, PDGF, EPO, CA9 and CXCR4, known to be important in metastatic processes [10, 11]. In addition to its destabilizing effect on HIF1/2α, pVHL is also involved in the recruitment of many effector proteins to regulate a variety of cellular processes including microtubule stability, activation of p53, neuronal apoptosis, cellular senescence and aneuploidy, ubiquitination of RNA polymerase II and regulation of NFkB activity [12].

*VHL* has been shown to be affected in more than 80 % of the ccRCC cases, either by allelic deletion, promoter methylation (19 %), or mutations (70–80 %) [13, 14]. Given the multiple functions of pVHL the inactivation of *VHL* is a critical point in the initiation of tumor formation in the context of ccRCC [15–17].

To date, controversial data exist about correlations between *VHL* mutations and pathological parameters, overall and disease-free survival [14, 18–22].

Whereas frameshift and nonsense mutations highly likely abrogate pVHL function, the effects on pVHL stability and binding ability of missense mutations occurring in about 25 % of ccRCC patients are rather unclear. Such mutations may not, partly or fully affect interacting functions of pVHL [23], which subsequently influence differently biological pathways involved in tumor carcinogenesis [24–31]. Evidence of mutant *VHL* expression at the RNA level [32, 33] as well as at the protein level [34, 35] was described in other studies. Although pVHL mutant forms tend to rapidly degrade, they still may exhibit partial function [31].

We therefore hypothesize that missense mutations exert different impact on the binding capability of pVHL targets and its pathways which may lead to diverse tumor aggressiveness and response to treatment. As treatments currently used in clinics for the metastatic disease are mostly anti-angiogenic tyrosine-kinase inhibitors targeting VEGFR and PDGFR to counter the upregulation of HIF caused by inactivation of *VHL*, it is of considerable interest to improve our knowledge on the additional HIF non-related pathways affected by *VHL* mutations.

In this study, we investigated the *VHL* mutation status in a cohort of 360 patients with sporadic ccRCC. We particularly focused on missense mutations and their potential biological effects on the pathways regulated by pVHL’s interactors as well as their impact on anti-angiogenic treatment response. The identification of ccRCC based on the pathways potentially affected by *VHL* missense mutations may be important for selecting appropriate targeted therapies.

## Methods

### Patients and tissue specimens

To a previously described collection of 256 formalin-fixed, paraffin-embedded (FFPE) tissue samples of patients with sporadic ccRCC [23], 90 additional cases from the University Hospital of Zürich and 14 from the Clinical Division of Oncology and Cancer Centre, Medical University of Vienna, Austria, were reviewed by one pathologist (H.M.). The tumors were graded according to the classification of the World Health Organization [4]. The median age of the patients was 64 years. Tumor stage and Fuhrman grade of the tumors were unknown for 14 patients. The cohort consisted of 147 (42.8 %) pT1, 31 (9 %) pT2, 160 (46.5 %) pT3 and 8 (2.3 %) pT4 ccRCC. There were 11 (3.2 %) grade I, 105 (30.5 %) grade II, 144 (41.9 %) grade III and 86 (25 %) grade IV tumors (see also Table 1). This study was approved by the cantonal commission of ethics of Zurich (KEK-ZH-nos. 2011–72 and 2013–0629). Areas that contained at least 75 % tumor cells were directly marked on the HE section of each tumor and considered for punching.

**Table 1:** Fuhrman grade, tumor stage and *VHL* mutation type in 346 ccRCC patients

	Fuhrman *n* (%)		Tumor stage (pT) *n* (%)	
*VHL* status	*1 + 2*	*3 + 4*	*1 + 2*	*3 + 4*
Nonsense	6 (21.4)	22 (78.6)	15 (53.6)	13 (46.4)
Frame shift	43 (44.8)	53 (55.2)	51 (53.1)	45 (46.9)
Missense	21 (26.9)	57 (73.1)	45 (56.3)	35 (43.8)
In frame	6 (35.3)	11 (64.7)	6 (37.5)	10 (62.5)
Splice site	3 (20)	12 (80)	9 (56.3)	7 (43.8)
Wild-type	37 (33)	75 (67)	52 (47.3)	58 (52.7)

Tumor stage or grade information was not available for 14 patients

Combined Fuhrman grades: 1 + 2 = low grade, 3 + 4 = high grade tumors

Combined tumor stages: 1 + 2 = organ-confined, 3 + 4 = metastatic

Thirty patients were treated with at least one of the following anti-angiogenic drugs: Sunitinib, sorafenib, pazopanib and bevacizumab. Tumor response was evaluated according to the RECIST criteria [36] and was classified into three types of response: progressive disease, stable disease and regressive disease (partial and complete remission) (data provided by Dr. Axel Mischo, Department of Oncology, University Hospital Zürich). The details of the treatments are shown in Table 3.

### DNA extraction and *VHL* sequencing

Total DNA was extracted from 3 to 4 tissue cylinders (diameter 0.6 mm) punched from each FFPE block and processed following the Qiagen DNeasy Blood & Tissue Kit (Qiagen, Germany) or the Maxwell® 16 FFPE Tissue LEV DNA Purification Kit (Promega corporation,USA).

The first 162 base pairs of *VHL* are rarely mutated and were excluded from sequence analysis [15]. The primers used for amplification were 5’-agagtccggcccggaggaact-3’ forward, 5’-gaccgtgctatcgtccctgc-3’ reverse for exon 1, 5’-accggtgtggctctttaaca-3’ forward and 5’-tcctgtacttaccacaacaacctt-3’ reverse for exon 2, and 5’-gagaccctagtctgtcactgag-3’ forward and 5’-tcatcagtaccatcaaaagctga-3’ reverse for exon 3. The forward and reverse DNA sequences overlap and cover the *VHL* sequence excluding the first 162 base pairs (Additional file 1). Sequencing was performed as described previously [23]. The sequences were aligned and compared to the NCBI sequence AF010238 using the informatics tool Sequencher (Sequencher® version 5.3 sequence analysis software, Gene Codes Corporation, Ann Arbor, MI USA, [37]). All *VHL* mutations were validated by a second independent PCR and sequence analysis.

### *In silico* analysis of *VHL* missense mutants

The effect of missense mutation on the stability of pVHL and its potential association to the disease were predicted *in silico* using the program Site Directed Mutator (SDM) [38]. The crystal structure of pVHL was isolated from VCB complex 1 lm8.pdb crystal structure (Piccolo database) and uploaded into the program to calculate the thermodynamic change (ddG) occurring after modification of one amino acid according to the main chain conformation, solvent accessibility and hydrogen bonding class. The missense mutations were then classified as follows:

- ddG < −2.0: highly destabilizing and disease-associated
−2.0 ≤ ddG < − 1.0: destabilizing
−1.0 ≤ ddG < −0.5: slightly destabilizing
−0.5 ≤ ddG ≤ 0.5: neutral
- 0.5 < ddG ≤ 1: slightly stabilizing
- 1.0 < ddG ≤ 2: stabilizing
- ddG > 2.0: highly stabilizing and disease-associated

The mapping of pVHL’s interactors binding domains has been adapted from Leonardi et al. [7].

### Statistics

A two-tailed Chi-Square statistics test with one degree of freedom was used for all the statistical tests in this study. Preferentially mutated codons of *VHL* were determined by calculating observed and expected frequencies of 88 out of 89 missense mutations.

## Results

### *VHL* mutation types, mutation sites, tumor stage and grade distribution

**Fig. 1:**
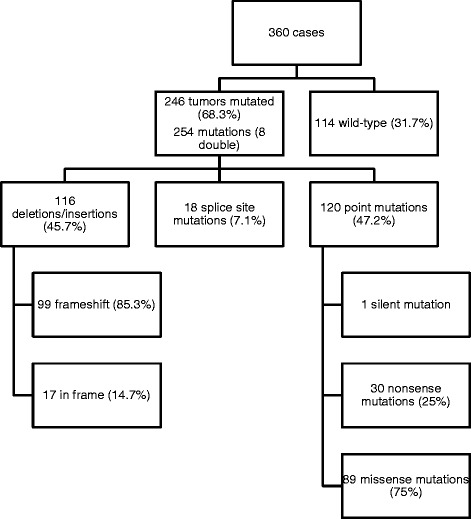
*VHL* sequence analysis of 360 ccRCC with frequencies of mutated tumors, total number of *VHL* mutations (including double mutations) as well as *VHL* mutation types. Deletions/Insertions were grouped into frameshift and in frame mutations; Point mutations were grouped into silent, nonsense and missense mutations

Two hundred forty-six of 360 (68.3 %) sequenced ccRCC were mutated. Eight of these tumors had two mutations. The frequencies of the *VHL* mutation types are illustrated in Fig. 1.

**Fig. 2:**
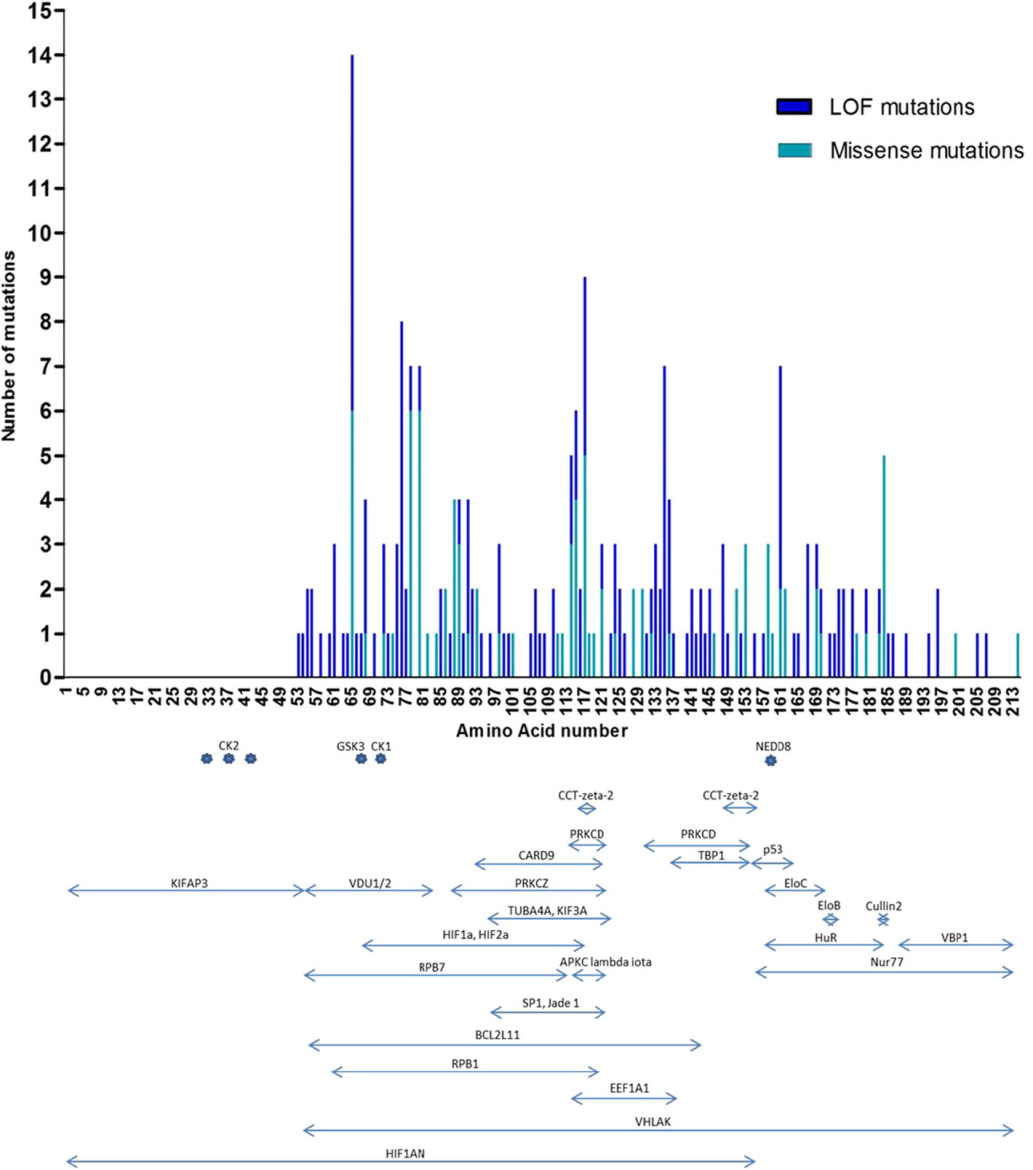
Frequencies of *VHL* LOF (loss of function; blue) and missense mutations (cyan), mutation sites and affected binding domains of pVHL’s interactors. Note: the first 162 base pairs (54 amino acids) were not sequenced

Since deletions, insertions, splice site mutations and nonsense mutations most likely abrogate most if not all pVHL functions, they were referred to as loss of function (LOF) mutations. An overview of *VHL* LOF and missense mutation sites in the pVHL sequence and the affected binding domains of pVHL’s interactors are shown in Fig. 2.

*VHL* mutation frequencies were similar in organ-confined pT1/2 and metastasizing pT3/4 ccRCC. There was no correlation between the number of mutations and stage or grade (Table 1). Additional information of the ccRCC specimens and the 256 mutations is given in Additional file 2: Table S1 and Additional file 3: Table S2.

### *VHL* mutation hotspots

A closer look at the mutation sites within the protein revealed that some codons were more frequently mutated than others. Fourteen mutations (5.5 %) were located at Ser65, nine (3.5 %) at Trp117, 8 (3.1 %) at Phe76, 7 (2.8 % each) at Asn78, Ser80, Leu135, and Arg161, 6 (2.4 %) at His115, and 5 mutations were at Gly114 and Leu184 (2 % each).

The codons that were most often affected by missense mutations were Ser65, Asn78, Ser80 (six mutations each, 6.7 %), Trp117 and Leu184 (five mutations each, 5.6 %). Codons Phe76 and Leu135 showed only LOF mutations.

### Preferentially affected binding domains of pVHL interactors

We next assigned 88 of 89 missense mutations to the putative binding domains of 32 pVHL interactors. One missense mutation in the stop codon was excluded from this analysis. As expected, large binding domains of interacting partners covering more than 60 amino acids of pVHL showed relative high frequencies of mutations. Between 44 and 100 % of the missense mutations were located in the VHLAK (100 %), HIF1AN (77.3 %), BCL2L11 (70.5 %), RPB1 (60.2 %), and RPB7 (44.3 %) binding domains. Notably, about half of the missense mutations (45/88, 51.1 %) resided in the HIF1α and HIF2α (EPAS1) binding domain comprising 51 amino acids.

Between 20 and 35 % of the missense mutations were located in the binding domains of PRKCD, VDU1/2, PRKCZ, EEF1A1, Nur77 and CARD9 (25–60 amino acids). The frequencies of missense mutations found in the smaller binding domains (9–28 amino acids) of JADE1, SP1, KIF3A, TUBA4A, HuR, aPKC-λ/ι, TBP1, CCT-ζ-2, EloC and p53 ranged between 8 and 23 %. All interactors, related pathways and binding domains affected by mutations are listed in Table 2.

**Table 2:** List of interactors and binding domains, number of missense mutations, comparison observed/expected frequency, and pathway affected

Name of the interactor	pVHL AA involved	Missense mutations count N (%)	Frequency of observed missense mutations compared to expected	*p*-value	Pathway of the interactor
CK2	S33, S38, S43	0 (0)	lower	ns	Protein amino acid phosphorylation
GSK3	S68	1 (1.1)	1.8X higher	ns	Wnt signaling pathway
CK1	S72	1 (1.1)	1.8X higher	ns	Wnt signaling pathway
NEDD8	K159	1 (1.1)	1.8X higher	ns	Ubl conjugation pathway
KIFAP3	1–54	0 (0)	lower	ns	Microtubule-based movement
HIF1αN	1–155	68 (77.3)	1.2X higher	**	HIF1α pathway
VDU1/USP33	54–83	22 (25)	1.3X higher	ns	Ubl conjugation pathway
VDU2/USP20	54–83	22 (25)	1.3X higher	ns	Ubl conjugation pathway
RPB7	54–113	39 (44.3)	1.2X higher	ns	Regulatory RNA pathways
VHLAK	54–213	88 (100)	equal	not applicable	Apoptosis
BCL2L11	55–143	62 (70.5)	1.3X higher	**	Apoptosis
HIF1α	67–117	45 (51.1)	1.6X higher	***	Hif1_tf pathway
EPAS1 (HIF2α)	67–117	45 (51.1)	1.6X higher	***	Vegfr1_2 pathway
RPB1	60–120	53 (60.2)	1.6X higher	***	Regulatory RNA pathways
PRKCZ	87–122	30 (34.1)	1.5X higher	**	Antiapoptosis, intracellular Signaling
CARD9	92–121	22 (25)	1.3X higher	ns	NFKB and MAPK signalling
TUBA4A	95–123	20 (22.7)	1.3X higher	ns	MT stabilization and dynamic cell polarity
KIF3A	95–123	20 (22.7)	1.3X higher	ns	Hedgehog_gli pathway
SP1	96–122	20 (22.7)	1.3X higher	ns	TGF-beta signaling pathway
JADE1	96–122	20 (22.7)	1.3X higher	ns	Apoptosis
PRKCD	113–122, 130–154	26 (29.5)	1.4X higher	ns	Regulation of receptor activity, senescence
aPKC-λ/ι	114–122	16 (18.2)	3.2X higher	***	Signalling by NGF
EEF1α1	114–138	23 (26.1)	1.7X higher	**	Protein biosynthesis
CCT-ζ-2	116–119, 148–155	13 (14.8)	2X higher	**	Chaperone-mediated protein complex assembly
TBP1	136–154	7 (8)	1.5X lower	ns	Signaling by Wnt, DNA Replication, Apoptosis
p53	154–163	8 (9.1)	1.5X higher	ns	Apoptosis
Nur77	155–213	20 (22.7)	1.6X lower	**	MAPK and NGF signaling pathways
EloC	157–171	11 (12.5)	1.3X higher	ns	Ubl conjugation pathway
HuR (RNA binding protein)	157–184	19 (21.6)	1.2X higher	ns	mRNA stabilization
EloB	170–174	1 (1.1)	2.8X lower	ns	Ubl conjugation pathway
Cullin2	181–184	6 (6.8)	2.7X higher	**	Ubl conjugation pathway
VBP1	187–213	1 (1.1)	14.9X lower	***	Morphogenesis

14 splice site mutations and a frameshift mutation for which the position of the affected amino acid cannot be determined and the missense mutation c.642 A > C/ p.X214Cys are excluded from this table. p-value summary: *P*-value: * < 0.05, ** < 0.01, *** < 0.001, ns “not significant”

Missense mutations which preferentially affected binding domains were identified by comparing the observed number with the expected number of mutation and by normalizing for each binding domain based on their amino acid length. As the first 54 amino acids of pVHL were not covered by Sanger sequencing, the expected number of missense mutation per codon was 0.55. We found that the binding domains showing significantly higher rates of missense mutations were for pVHL interactors HIF1AN, BCL2L11, HIF1α, HIF2α, RPB1, PRKCZ, aPKC-λ/ι, EEF1A1, CCT-ζ-2, and Cullin2. pVHL binding partners with involved pathways and the ratio of observed versus expected frequency of missense mutations are shown in Table 2. Additional information on pVHL binding partners is given in Additional file 4.

### *VHL* missense mutations and pVHL stability

Eighty-eight missense mutations were analyzed *in silico* using the program SDM to determine the protein thermodynamic change (ddG) triggered by those mutations. In this context, ddG is an indicator of pVHL stability and suggests whether or not a missense mutation causes deleterious functional impact and is associated with disease.

A large proportion of the *VHL* missense mutations (60/88, 68 %) were predicted to destabilize the resulting protein (ddG < −0.5), eleven mutations (11/88, 12.5 %) had a neutral effect (−0.5 < ddG < 0.5), and 17 had a stabilizing effect (17/88, 19.3 %) on pVHL. Thirty-three of 88 (37.5 %) missense mutations were highly destabilizing and only 2 (2.3 %), were highly stabilizing, suggesting that about 40 % of *VHL* missense mutations were predicted to cause protein malfunction (ddG < −2 and ddG > 2 respectively). *VHL* missense mutations and their predicted effects on pVHL stability and association with disease are listed in Additional file 5: Table S3.

By focusing on the HIF1/2α binding domain (amino acids 67–117) and the remaining parts of the protein (amino acids 54–66, 118–213) we observed significantly more missense mutations in the HIF1/2α binding domain than expected (43/88 observed, 28/88 expected (*p*-value <0.0001). However, the frequency of destabilizing mutations (ddG < −0.5) in the HIF1/2α binding domain (32/45, 71.1 %) was similar to that seen for the remaining parts of the protein (28/43, 65.1 %).

**Fig. 3:**
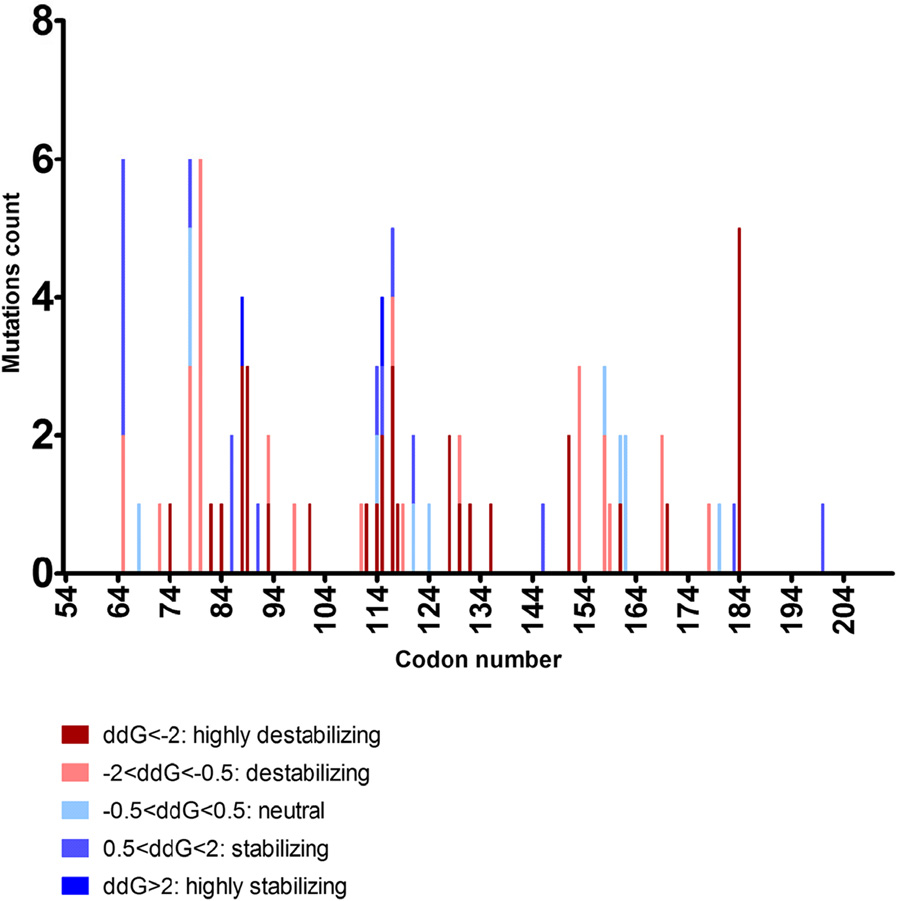
Distribution and frequency of *VHL* missense mutations and their predicted effects on pVHL stability using the program Site Directed Mutator (SDM) [38]

Notably, all of the hotspot missense mutations found in codons Trp117 and Leu184 were destabilizing and 3 out of 5 and 5 out of 5 mutations, respectively, were predicted to cause protein malfunction. In addition, all missense mutations in codon Ser80 destabilize pVHL, codon Ser65 had 3 destabilizing and 3 stabilizing mutations, and codon Ser65 had 2 destabilizing and 4 stabilizing mutations. The sites of all missense mutations are shown together with their stability prediction in Fig. 3.

### pVHL mutations and treatment response

After surgical resection of the primary tumor, 30 patients from the cohort were treated with anti-angiogenic drugs that are currently used in clinics for patients with metastatic ccRCC. These patients were subdivided into three groups according to response to therapy: progressive, stable and regressive disease. Treatment administered, response, *VHL* mutation status, tumor stage and grade are listed in Table 3.

**Table 3:** Treatment, response, and *VHL* mutation status of the patients treated with anti-angiogenic therapies

Mutation	Mutation consequence	Functionality prediction	Interacting partners	Disease progression status	Treatment	pT stage	Fuhrman grade
c.163delG/p.Glu55ArgfsX11	fs	LOF		PD	Pazopanib > Everolimus	3	3
c172delC/p.Arg58GlyfsX9	fs	LOF		PD	IFNa > Pazopanib		
c.194C > T/p.Ser65Leu	missense	stabilizing	HIF1αN/VDU1/USP33/VDU2/USP20/RPB7/VHLAK/BCL2L11/RPB1	PD	Sunitinib		
c.240 T > A/p.Ser80Arg	missense	destabilizing	HIF1αN/VDU1/USP33/VDU2/USP20/RPB7/VHLAK/BCL2L11/HIF1α/EPAS1/RPB1	PD	IFNa > Sorafenib	1	
c. 262 T > A/p.Trp88Arg	missense	highly destabilizing	HIF1αN/RPB7/VHLAK/BCL2L11/HIF1α/EPAS1/RPB1/PRKCZ	PD	Sunitinib	3	3
c.268_273del/p.Asn90_Phe91del	in frame	LOF		PD	Sunitinib		3
c.IVS1 + 1G > A (c.340 + 1G > A)	splice mut	LOF		PD	Sorafenib > Sunitinib > Everolimus		
c.345_364del/p.Leu116ArgfsX9	fs	LOF		PD	IFNa > Sorafenib		
c.349delT/p.Trp117GlyfsX42	fs	LOF		PD	Sunitinib	3	3
c.484 T > C/p.Cys162Arg	missense	neutral	VHLAK/p53/Nur77/EloC/HuR	PD	Sunitinib > Sorafenib > Everolimus > Pazopanib	3	3
c.497_505del9/p.Arg167ValdelSerLeu	in frame	LOF		PD	Sunitinib	3	3
C.580_583delinsAA/p.Val194LysfsX61	fs	LOF		PD	Sunitinib > Sorafenib		
c.586A > T/p.Lys196X	nonsense	LOF		PD	Sunitinib > Sorafenib > Everolimus	1	
	wild-type	wild-type		PD	Pazopanib	3	4
	wild-type	wild-type		PD	Sunitinib > Pazopanib > Sorafenib > Everolimus	4	3
	wild-type	wild-type		PD	Sunitinib	2	3
c.161_162delTG/p.Met54ArgfsX77	fs	LOF		SD	Sunitinib	3	4
c.203C > A/p.Ser68X	nonsense	LOF		SD	Sorafenib > Pazopanib > Everolimus	3	4
c.327insA/p.His110ProfsX22	fs	LOF		SD	Sunitinib > Sorafenib	1	4
c.IVS1 + 2 T > A (c.340 + 2 T > A)	splice mut	LOF		SD	Pazopanib > Axitinib	3	3
c.345insC/p.Leu116ProfsX15	fs	LOF		SD	Bevacizumab > IFNa > Pazopanib	3	4
c.350delG/p.Trp117CysfsX42	fs	LOF		SD	IFNa/Bevacizumab	1	3
c.481C > T/p.Arg161X	nonsense	LOF		SD	Sorafenib	2	2
	wild-type	wild-type		SD	Sunitinib > Sorafenib > Everolimus		
c.167_168delCC/p.Ala56GlyfsX75	fs	LOF		RD	Sorafenib	3	1
c.227_229del3/p.Phe76del	in frame	LOF		RD	Pazopanib > Sunitinib	1	3
c.340G > T/p.Gly114Cys	missense	neutral	HIF1αN/VHLAK/BCL2L11/HIF1α/EPAS1/RPB1/PRKCZ/CARD9/TUBA4A/KIF3A/SP1/JADE1/PRKCD/aPKC-λ/ι/EEF1A1	RD	IFNa > Bevacizumab	2	3
*c.383 T > C/p.Leu128Pro* ^*a*^	*missense*	*higly destabilizing*	*HIF1αN/VHLAK/BCL2L11/EEF1A1*	*RD*	*Pazopanib*	*3*	*4*
*c.430G > T/p.Gly144X* ^*a*^	*nonsense*	*LOF*		*RD*	*Pazopanib*	*3*	*4*
c.458 T > C/p.Leu153Pro	missense	destabilizing	HIF1αN/VHLAK/PRKCD/CCT-ζ-2/TBP1	RD	Sunitinib	2	3
	wild-type	wild-type		RD	Pazopanib > Everolimus	3	3

*PD* progressive disease, *SD* Stable disease, *RD* Regressive disease, *LOF* loss-of-function, *fs* frameshift

^*a*^one patient with two mutations

The proportion of responders (stable + regressive disease) was 52.6 % for the LOF (10/19), 33.3 % for the missense mutations (2/6), and 40 % for the wild-type *VHL* (2/5).

There was no correlation between disease progression status, tumor stage, grade, VHL mutation types and specific treatments.

## Discussion

It is widely accepted that in almost all ccRCC both *VHL* alleles are inactivated by chromosome 3p loss, mutation and hypermethylation [13, 14, 39]. In contrast to frameshifts, nonsense codons and alteration of splice sites, which highly likely cause loss of function of pVHL in about 50 % of these tumors, the consequences of *VHL* missense mutations present in 25 % may significantly vary. A detailed and comprehensive investigation of such mutations in this context can hardly be found in the literature. The goal of our study was therefore to sequence the *VHL* tumor suppressor gene in 360 ccRCC patients and characterize missense mutations by focusing on preferentially affected sites in the gene and their potential consequences on pVHL function and its binding partners.

Intratumoral heterogeneity is a common feature of most cancers and represents a big challenge for molecular diagnostics. To avoid any false negative artifacts we paid attention to analyze the *VHL* sequence of one paraffin embedded ccRCC tissue block that contained at least 70 % tumor cells. The high mutation rate in our ccRCC cohort confirmed previous results showing that *VHL* alteration is rather independent of heterogeneity and ubiquitously present in ccRCC [40]. In cases with intratumoral heterogeneity related to *VHL*, own studies have shown the presence of de novo *VHL* mutations [41]. Minor populations of tumor cells with *VHL* mutations are extremely rare [40]. We therefore conclude that most non-mutated tumors were in fact *VHL* wild type and that the use of more than one FFPE block to analyze one tumor would have not influenced significantly our results. Next generation sequence analysis of additional genes demonstrated that intratumoral heterogeneity increases with the number of tumor regions sequenced [40, 42]. The relevance of molecular findings in other genes may thus be more reliable if several blocks are used. The analysis of several areas in one tumor could allow identifying subclonal driver mutations in other genes that may be responsible for drug resistance.

The frequency of *VHL* mutations found in about 70 % of the patients was comparable to previously published data [16]. There was no correlation with *VHL* mutation types and the prognostic parameters tumor stage and grade, which is consistent with previous studies [16, 20, 43]. Although most of the *VHL* mutations were private, we found several hotspot mutations in our cohort. Between 5 and 14 mutations affected codons Ser65, Phe76, Asn78, Ser80, Gly114, His115, Trp117, Leu135, Arg161 and Leu184. Interestingly, approximately one third of the 88 missense mutations occurred at codons Ser65, Asn78, Ser80, Trp117 and Leu184 (5–6 mutations per codon). Those missense mutations have already been described in the *VHL* mutations database-UMD [44] and in the COSMIC database for ccRCC [45] where they represent about 10 % of all *VHL* mutations. This frequency is consistent with our finding (28/256, 10.9 %) and confirms the quality of the sequencing data obtained from our patient cohort.

In addition to the hotspot missense mutations, we also noticed considerable discrepancies between the expected and observed number of missense mutations which particularly affected the binding domains of 10 of 32 pVHL targets. Significant more missense mutations than expected were seen in binding domains specific for HIF1AN, BCL2L11, HIF1α, HIF2α, RPB1, PRKCZ, aPKC-λ/ι, EEF1A1, CCT-ζ-2, and Cullin2. Apart from HIFα, most of these proteins are mainly involved in apoptosis (BCL2L11, aPKC-λ/ι), transcriptional regulation (RPB1, PRKCZ) and ubiquitin ligation (CCT-ζ-2, Cullin2). Some of these missense mutations may exert pleiotropic effects on different pathways. This was recently shown with the mutants Phe81Ser and Arg167Gln which cause partial abrogation of VBC complex interactions and fail to downregulate HIF1/2α. Simultaneously, they also lead to enhanced anti-apoptosis signaling and weaken the assembly of RNA Polymerase II complex and protein ubiquitination signaling pathway [46]. Notably, the binding sites for aPKC-λ/ι, CCT-ζ-2, and Cullin2 were the most affected ones and may thus represent potential drug targets alternatively to HIF. For example, disruption of pVHL binding leads to subsequent ubiquitination of aPKC-λ/ι, which in turn deregulates JunB expression and promotes tumor progression in VHL disease-related pheochromocytoma. Uncontrolled expression of JunB may also be important in ccRCC as JunB was found to be upregulated in sporadic, pVHL inactivated, ccRCC [47, 48]. Moreover, *VHL* mutations were shown to impair the interaction with pVHL and CCT-ζ-2 which, consequently, caused improper folding of the VBC complex [25, 49]. Given the function of Cullin2 a default in VBC complex formation may also be expected from disrupted binding of pVHL with this protein. Interestingly, the binding domain for VBP1 located at the 3’ end of *VHL* exon 3 seems to be spared from mutations. VBP1 functions as a chaperone protein and may play a role in the transport of pVHL from the perinuclear granules to the nucleus or cytoplasm [50]. The strikingly low frequency of mutations (15 times lower than expected) in this region of *VHL* may reflect the importance of sustaining accurate pVHL trafficking in ccRCC. This is supported by a previous report showing that ccRCC with pVHL expression in both nuclear and cytoplasmic compartments had a better prognosis [34].

The effects of missense mutations on protein stability were determined *in silico* by calculating the thermodynamic change caused by one missense mutation. The tool for determining protein stability was proven powerful with mutations predicted to be highly destabilizing leading to both faster degradation of pVHL and stabilization of HIF1/2α [23]. Based on this observation it is conceivable that those mutations are critical for most if not all binding partners of pVHL.

In addition to their potential influence on pVHL function we also attempted to further characterize the 88 missense mutations with regard to their tumorigenic potential. We used the Symphony classification system that allows subclassifying *VHL* missense mutations in VHL disease patients according to their risk of developing ccRCC [51]. Among the 88 missense mutations, 61 (80 %) were classified by Symphony as high risk of developing ccRCC. We conclude that most of the missense mutations, even those with neutral or mild impact on pVHL stability as predicted by SDM, may have strong tumorigenic potential. Notably, only two of the remaining 17 missense mutations were highly destabilizing mutations (Ile151Ser and His115Leu) and classified as low risk of ccRCC.

Current therapeutic strategies for ccRCC focus on Tyrosine Kinase Inhibitors (such as sunitinib, sorafenib, pazopanib, axitinib) or other anti-angiogenic drugs (i.e. bevacizumab) to counteract VEGF/ PDGF upregulation in *VHL* mutated tumors with accumulated HIF1/2α [52]. Treatment with Sunitinib as the most commonly used targeted therapy show mainly partial response in 31 % of the patients with metastatic ccRCC [5]. It is tempting to speculate that the response rate of ccRCC patients may be linked to the *VHL* mutation type present in a tumor. We therefore analyzed follow-up data of 30 ccRCC patients with known *VHL* mutation status who were treated with anti-angiogenic drugs. Fifty-three percent of the patients with LOF, 33 % with missense mutations, and 40 % wild-type responded to the treatment (regressive or stable disease). No significant association was seen between *VHL* mutation status and response to treatment in our cohort, although a higher response rate in patients with LOF compared to wild-type or missense mutations has been described in a larger study [22].

Using novel high throughput sequencing platforms novel driver genes were identified in ccRCC. Frequent alterations were found in the genes *SETD2*, *BAP1*, and *PBRM1*, which are all located on chromosome 3p in close proximity to *VHL* [53]. Mutations in the two latter genes seem to be linked to enhanced cell proliferation, tumor aggressiveness and patient outcome. Twenty percent of ccRCC have mutations in *MTOR*, *TSC1*, *PIK3CA*, and *PTEN* and indicates that deregulated mTOR pathways may also be critical in this tumor subtype. Interestingly, up to 5 % of ccRCC with intact *VHL* are characterized by loss of heterozygosity of 8q21 and mutations in *TCEB1*, which is located in this chromosomal region. *TCEB1* encodes Elongin C, a member of E3 ubiquitin protein ligase that binds to pVHL. The new 2016 WHO classification has not yet recognized RCC with *TCEB1* mutations as own tumor entity, but included such tumors in the category of emerging entities [54, 55]. A future RCC terminology could be based even more on such molecular findings. In ccRCC, loss of function of either pVHL or Elongin C may result in HIF stabilization. In search for better individualized therapies of ccRCC, these discoveries suggest the need to open a new consensus on terminology, cut-offs and genetic classification when dealing with the analytical and interpretative phases of molecular findings.

## Conclusions

**Fig. 4:**
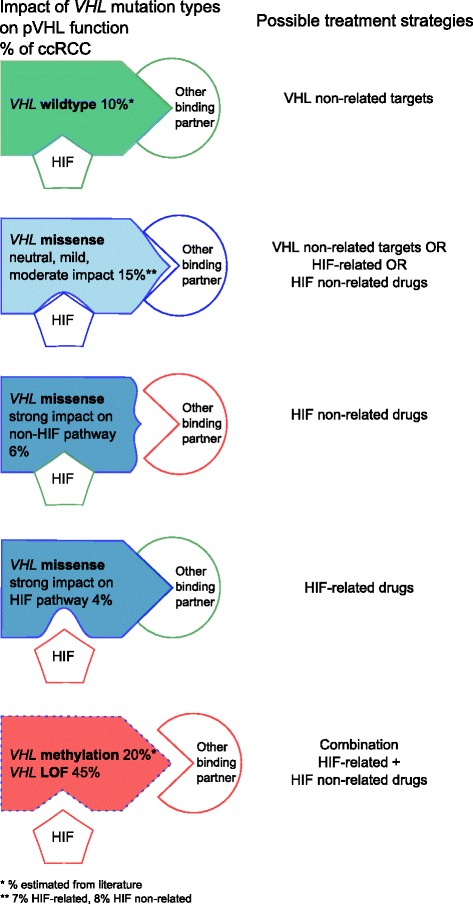
Impact of *VHL* mutation type on pVHL function and possible treatment strategies

In summary, our *VHL* sequence analysis of 360 ccRCC revealed pVHL binding sites which are preferentially altered by missense mutations. In contrast to LOF mutations which probably influence most of the pVHL regulated pathways, missense mutations may rather deregulate only single or few of those pathways. Moreover, about 15 % of ccRCC patients having missense mutations with no, mild or only moderate impact on pVHL function even may have fully or at least partially functional pVHL. As a consequence, pVHL may retain full ability to degrade HIF1/2α but lose its binding ability to other interactors and vice versa. We therefore hypothesize that the relatively low response rate to anti-angiogenic drugs may be explained by the multipurpose nature of pVHL and the manifold effects on pathways caused by the different mutation types. Patients with *VHL* missense mutation may rather benefit from targeted therapies than patients with LOF mutations (Fig. 4). For *VHL* wild-type tumors, other therapy modalities aiming at pVHL non-related pathways controlled by tumor suppressors such as *PBRM1*, *SETD2* or *BAP1* may be more appropriate than the common anti-angiogenic treatment [56–64].

## Additional files

Additional file 1: VHL sequence analysis. (DOCX 28 kb) Additional file 2: Table S1. VHL mutation type, Fuhrman grade and tumor stage of 360 ccRCC. (XLSX 11 kb) Additional file 3: Table S2. List of all VHL mutations. (XLSX 21 kb) Additional file 4: Supplementary information on binding partners. (DOCX 63 kb) Additional file 5: Table S3. List of missense mutations, stability and disease association prediction. (XLSX 16 kb)

## Abbreviations

ccRCC: Clear Cell Renal Cell Carcinoma
FFPE: Formalin-fixed, Paraffin-embedded
HIF: Hypoxia-inducible factor
LOF: Loss-of-function
pVHL: VHL protein
SDM: Site-directed-mutator.
