# Supplementary material for: Characterization of VHL missense mutations in sporadic clear cell renal cell carcinoma: hotspots, affected binding domains, functional impact on pVHL and therapeutic relevance
Source: BMC Cancer. 2016 Aug 17;16:638. doi: 10.1186/s12885-016-2688-0 (PMC4987997; doi:10.1186/s12885-016-2688-0)
Supplement: Additional file 1: — VHL sequence analysis. (DOCX 28 kb) [file 12885_2016_2688_MOESM1_ESM.docx]

**Supplementary S1: *VHL* sequence analysis**

1 2 3 4 5 6 7 8 9 10 11 12 13 14 15 16 17 18 19 20

M P R R A E N W D E A E V G A E E A G V

1 ***a****tg ccc cgg* ***a****gg gcg gag a****a****c tgg gac ga****g*** *gcc gag gta* ***g****gc gcg gag g****a****g gca ggc gtc* 60

21 22 23 24 25 26 27 28 29 30 31 32 33 34 35 36 37 38 39 40

E E Y G P E E D G G E E S G A E E S G P

61 *gaa gag tac* ***g****gc cct gaa g****a****a gac ggc gg****g*** *gag gag tcg* ***g****gc gcc gag g****a****g tcc ggc ccg* 120

41 42 43 44 45 46 47 48 49 50 51 52 53 54 55 56 57 58 59 60

E E S G P E E L G A E E E M E A G R P R

121 *gaa gag tcc* ***g****gc ccg gag g****a****a ctg ggc gc****c*** *gag gag gag* ***a****tg* gag gcc g**g**g cgg ccg cgg 180 Exon 1

61 62 63 64 65 66 67 68 69 70 71 72 73 74 75 76 77 78 79 80

P V L R S V N S R E P S Q V I F C N R S

181 ccc gtg ctg **c**gc tcg gtg a**a**c tcg cgc ga**g** ccc tcc cag **g**tc atc ttc t**g**c aat cgc agt 240

81 82 83 84 85 86 87 88 89 90 91 92 93 94 95 96 97 98 99 100

P R V V L P V W L N F D G E P Q P Y P T

241 ccg cgc gtc **g**tg ctg ccc g**t**a tgg ctc aa**c** ttc gac ggc **g**ag ccg cag c**c**c tac cca acg 300

101 102 103 104 105 106 107 108 109 110 11 112 113 114

L P P G T G R R I H S Y R G

301 ctg ccg cct **g**gc acg ggc c**g**c cgc atc ca**c** agc tac cga **g**gtacgggcccggcgcttaggcccga

cccagcagggacgatagcacggtctgaagcc

114 115 116 117 118 119 120

G H L W L F R

caccggtgtggctctttaacaacctttgcttgtcccgata**G**t cac ctt t**g**g ctc ttc aga 360

121 122 123 124 125 126 127 128 129 130 131 132 133 134 135 136 137 138 139 140

D A G T H D G L L V N Q T E L F V P S L

361 gat gca ggg **a**ca cac gat g**g**g ctt ctg gt**t** aac caa act **g**aa tta ttt g**t**g cca tct ctc 420 Exon 2

141 142 143 144 145 146 147 148 149 150 151 152 153 154 155

N V D G Q P I F A N I T L P V

421 aat gtt gac **g**ga cag cct a**t**t ttt gcc aa**t** atc aca ctg **c**ca ggtactgacgttttacttttta

aaaagataaggttgttgtggtaagtacaggataga

155 156 157 158 159 160

V Y T L K E

ctgagaccctagtctgtcactgagga**T**g tat a**c**t ctg aaa gag 480

161 162 163 164 165 166 167 168 169 170 171 172 173 174 175 176 177 178 179 180

R C L Q V V R S L V K P E N Y R R L D I

481 cga tgc ctc **c**ag gtt gtc c**g**g agc cta gt**c** aag cct gag **a**at tac agg a**g**a ctg gac atc 540 Exon 3

181 182 183 184 185 186 187 188 189 190 191 192 193 194 195 196 197 198 199 200

V R S L Y E D L E D H P N V Q K D L E R

541 gtc agg tcg **c**tc tac gaa g**a**t ctg gaa ga**c** cac cca aat **g**tg cag aaa g**a**c ctg gag cgg 600

201 202 203 204 205 206 207 208 209 210 211 212 213

L T Q E R I A H Q R M G D *stop

601 ctg aca cag **g**ag cgc att g**c**a cat caa cg**g** atg gga gat **t**gaagatttctgttgaaacttacact

gtttcatctcagcttttgatggtactgatgag

Grey: Exons sequenced;

Red: Primer sequences;

Italics: Base pairs excluded from sequence analysis;
