# Supplementary material for: Characterization of VHL missense mutations in sporadic clear cell renal cell carcinoma: hotspots, affected binding domains, functional impact on pVHL and therapeutic relevance
Source: BMC Cancer. 2016 Aug 17;16:638. doi: 10.1186/s12885-016-2688-0 (PMC4987997; doi:10.1186/s12885-016-2688-0)
Supplement: Additional file 4: — Supplementary information on binding partners. (DOCX 63 kb) [file 12885_2016_2688_MOESM4_ESM.docx]

**Preferentially altered binding domains**

HIF1AN (alias FIH1) is an inhibitor of the α subunit of HIF1 that interacts with pVHL and HIF1a to mediate repression of HIF1 transcriptional activity [1] by preventing HIF-1α from binding to p300/CBP [2].

BCL2L11 is an apoptosis facilitator leading to the expression of BIM(EL) protein which can be stabilized by *VHL* wild-type protein [3].

HIF1α is the alpha subunit of transcription factor hypoxia-inducible factor-1, which is formed by the association of an alpha and a beta subunit. HIF-1 is a regulator of cellular response to hypoxia that activates transcription of many genes for metabolism, angiogenesis and apoptosis for adaptation to hypoxia. One of pVHL major role is to facilitate the oxygen-dependent ubiquitination of HIF1 for proteasomal degradation, leading to downregulation of HIF target genes [4].

HIF2α is the alpha subunit of hypoxia-inducible factor-2, a transcription factor responding to hypoxia and involved in the induction of genes regulated by oxygen. As HIF1α, its ubiquitination is mediated by pVHL which acts as a downregulator of HIF2α [5].

RPB1 is the largest subunit of the RNA polymerase II complex that can be ubiquitinated by pVHL thus regulating its expression in RCC cells [6]. Levels of Rpb1 are significantly higher in RCC tumors compared with normal kidneys and RCC tumors with pVHL wild-type show higher levels of Rpb1 than tumors with *VHL* mutations [7].

PRKCZ is a serine/threonine kinase which is recruited by pVHL causing ubiquitination and degradation thus influencing cell polarity [8, 9].

aPKC-λ/ι is a tyrosine kinase member of the protein kinase C family and aPKC isotypes are involved in the regulation of cell growth and apoptosis and interact directly with the β-domain of pVHL [10].

EEF1A1 encodes the alpha subunit of the elongation factor-1 complex, which is responsible for the enzymatic delivery of aminoacyl tRNAs to the ribosome. This translation factor interacts specifically with the transcription-dependent nuclear export motif of *VHL*, mediating the nuclear export of pVHL [11].

CCT-ζ-2 is a molecular chaperone protein, member of the chaperonin containing TCP1 complex (CCT). CCT-ζ-2 mediates the proper folding and assembly of the VCB complex and some *VHL* mutations have been demonstrated to impair this interaction [12, 13].

Cullin2 is a negative regulator of cell cycle and associates with pVHL in the VBC complex [14, 15].

**Spared binding domains**

Nur77 is a nuclear transcription factor that promotes cancer cell growth when located in the nucleus or induces apoptosis when translocated to mitochondria. Nur77 indirectly stabilizes HIF-α by binding to pVHL, thus increasing HIF1α transcriptional activity [16].

VBP1 interacts with pVHL to form an intracellular complex. VBP1 is a chaperone protein, and pVHL plays a role in its transport from the perinuclear granules to the nucleus or cytoplasm but VBP1 implication in ccRCC remains unknown [17].

**Binding domains more affected than expected without reaching significance**

GSK3 is a glycogen synthase kinase that phosphorylates pVHL at position Ser68 thus negatively regulating microtubule stabilization by pVHL [18].

CK1 is a serine/threonine kinase involved in the regulation of the G1-checkpoint. CK1 phosphorylates pVHL at Ser72 which is a priming event to phosphorylation of pVHL’s Ser68 by GSK3 [18].

NEDD8 is an ubiquitin-like protein playing an important role in cell cycle control. NEDD8 association with pVHL prevents the formation of the VCB complex [19].

VDU1 is able to be ubiquitinated via a pVHL-dependent pathway for proteasomal degradation, and *VHL* mutations that disrupt the interaction between VDU1 and pVHL abrogate the ubiquitination of VDU1 [20, 21].

VDU2 can also be ubiquitinated and degraded in a pVHL-dependent manner, preventing it from rescuing HIF1α degradation by deubiquitination [21].

RPB7 is the seventh largest subunit of the RNA polymerase II complex. *VHL* protein facilitates its ubiquitination and proteasomal degradation and decreases its nuclear accumulation. pVHL can also suppress hsRPB7-induced VEGF promoter transactivation, mRNA expression and VEGF protein secretion [22].

CARD9 is an activator of BCL10 leading to NFKB activation and also acts as a positive regulator of apoptosis. VHL protein associates with CARD9 and promotes its phosphorylation by CK2, thus inhibiting its activation of NFkB [23].

TUBA4A is a member of the tubulin superfamily and one of the major components of microtubules. pVHL binds α-tubulin and stabilizes it, stabilizing microtubules [24-26].

KIF3A is a member of the kinesin protein family mediating pVHL’s interaction with microtubules [25].

SP1 is implicated in regulation of genes that control multiple cellular processes, including cell cycle, apoptosis, and DNA damage. pVHL inhibits sp1 interaction with PKC zeta and Sp1-dependent transcriptional regulation of VEGF expression and thus tumor angiogenesis [27].

JADE1 is involved in apoptosis and differentiation in epithelia and ubiquitinates βcatenin for degradation. This protein is stabilized by interaction with pVHL and this stabilization is VHL mutation-dependent [28-30].

PRKCD is a serine/threonine kinase involved in diverse cellular signaling pathways such as growth, apoptosis, and differentiation. pVHL blocks the interaction of PRKCD with IGF1R to decrease tumor progression [31].

p53 is a tumor suppressor protein responding to diverse cellular stresses such as DNA damage and hypoxia to regulate expression of target genes leading to cell cycle arrest, apoptosis, senescence, DNA repair, or changes in metabolism. Recently p53 has been shown to interact with pVHL. This interaction prevents p53 ubiquitination by the Mdm2 protein, therefore leading to p53 stabilization and transactivation of p53 target genes for apoptosis and cell cycle arrest [32-35].

EloC is a subunit of the transcription factor B composed of elongins A/A2, B and C and activating elongation by RNA polymerase II. EloC is also one component of the VCB complex for negative regulation of HIFs [36].

HuR is a RNA binding protein regulating gene expression that is highly expressed in many cancers including ccRCC where it is activated in the early tumor stages. HuR induces VEGF and IGF1R mRNA stabilization and pVHL interaction with HuR has been demonstrated to antagonize these effects [37-39].

**Binding domains less affected than expected without reaching significance**

CK2 is a protein involved in regulation of cell growth that phosphorylates the acidic domain of pVHL to stabilize its interaction with fibronectin [40].

TBP1 is a regulator of proteasome, ATPase subunit contributing to the E3 ubiquitin ligase function of the VHL protein. TBP-1 has been show to interact with the β-domain of pVHL and to form a complex with pVHL and HIF1α to promote HIF1α degradation [41].

KIFAP3A is kinesin-associated protein involved in pVHL subcellular mobility [42].

EloB is a subunit of the transcription factor B and one component of the VCB complex for negative regulation of HIFs [36].

**Binding domain with observed mutation frequency equaling expected frequency**

VHLAK, also known as zinc finger protein 197, is a regulatory and transcription factor involved in transcriptional regulation that acts as a negative regulator of HIF-1alpha transactivation. *VHL* protein recruits VHLAK to repress HIF-1alpha transcriptional activity and HIF-1alpha-induced VEGF expression [43].

1. Mahon, P.C., K. Hirota, and G.L. Semenza, *FIH-1: a novel protein that interacts with HIF-1α and VHL to mediate repression of HIF-1 transcriptional activity.* Genes & Development, 2001. **15**(20): p. 2675-2686.

2. Sirin, Y. and H. Pavenstadt, *FIH1 (factor inhibiting HIF-1) in the kidney: more than an oxygen sensor[quest].* Kidney Int, 2010. **78**(9): p. 836-837.

3. Guo, Y., M.C. Schoell, and R.S. Freeman, *The von Hippel-Lindau protein sensitizes renal carcinoma cells to apoptotic stimuli through stabilization of BIM(EL).* Oncogene, 2009. **28**(16): p. 1864-74.

4. Keefe, S.M., K.L. Nathanson, and W. Kimryn Rathmell, *The Molecular Biology of Renal Cell Carcinoma.* Seminars in Oncology, 2013. **40**(4): p. 421-428.

5. Kaelin, W.G., Jr., *The von Hippel-Lindau tumour suppressor protein: O2 sensing and cancer.* Nat Rev Cancer, 2008. **8**(11): p. 865-73.

6. Mikhaylova, O., et al., *The von Hippel-Lindau Tumor Suppressor Protein and Egl-9-Type Proline Hydroxylases Regulate the Large Subunit of RNA Polymerase II in Response to Oxidative Stress.* Molecular and Cellular Biology, 2008. **28**(8): p. 2701-2717.

7. Yi, Y., et al., *von Hippel-Lindau–Dependent Patterns of RNA Polymerase II Hydroxylation in Human Renal Clear Cell Carcinomas.* Clinical Cancer Research, 2010. **16**(21): p. 5142-5152.

8. Iturrioz, X., et al., *The von Hippel-Lindau tumour-suppressor protein interaction with protein kinase Cdelta.* Biochem J, 2006. **397**(1): p. 109-20.

9. Iturrioz, X. and P.J. Parker, *PKCζII is a target for degradation through the tumour suppressor protein pVHL.* FEBS Letters, 2007. **581**(7): p. 1397-1402.

10. Okuda, H., et al., *Direct Interaction of the β-Domain of VHL Tumor Suppressor Protein with the Regulatory Domain of Atypical PKC Isotypes.* Biochemical and Biophysical Research Communications, 1999. **263**(2): p. 491-497.

11. Khacho, M., et al., *eEF1A Is a Novel Component of the Mammalian Nuclear Protein Export Machinery.* Molecular Biology of the Cell, 2008. **19**(12): p. 5296-5308.

12. Feldman, D.E., et al., *Formation of the VHL–Elongin BC Tumor Suppressor Complex Is Mediated by the Chaperonin TRiC.* Molecular Cell, 1999. **4**(6): p. 1051-1061.

13. Feldman, D.E., et al., *Tumorigenic Mutations in VHL Disrupt Folding In Vivo by Interfering with Chaperonin Binding.* Molecular Cell, 2003. **12**(5): p. 1213-1224.

14. Kinoshita, K., et al., *Ternary complex formation of pVHL, elongin B and elongin C visualized in living cells by a fluorescence resonance energy transfer-fluorescence lifetime imaging microscopy technique.* FEBS J, 2007. **274**(21): p. 5567-75.

15. Kamura, T., et al., *VHL-box and SOCS-box domains determine binding specificity for Cul2-Rbx1 and Cul5-Rbx2 modules of ubiquitin ligases.* Genes & Development, 2004. **18**(24): p. 3055-3065.

16. Kim, B.-Y., et al., *Nur77 upregulates HIF-[alpha] by inhibiting pVHL-mediated degradation.* Exp Mol Med, 2008. **40**: p. 71-83.

17. Tsuchiya, H., T. Iseda, and O. Hino, *Identification of a Novel Protein (VBP-1) Binding to the von Hippel-Lindau (VHL) Tumor Suppressor Gene Product.* Cancer Research, 1996. **56**(13): p. 2881-2885.

18. Hergovich, A., et al., *Priming-Dependent Phosphorylation and Regulation of the Tumor Suppressor pVHL by Glycogen Synthase Kinase 3.* Molecular and Cellular Biology, 2006. **26**(15): p. 5784-5796.

19. Russell, R.C. and M. Ohh, *NEDD8 acts as a ‘molecular switch’ defining the functional selectivity of VHL*. Vol. 9. 2008. 486-491.

20. Li, Z., et al., *Ubiquitination of a Novel Deubiquitinating Enzyme Requires Direct Binding to von Hippel-Lindau Tumor Suppressor Protein.* Journal of Biological Chemistry, 2002. **277**(7): p. 4656-4662.

21. Li, Z., et al., *Identification of a deubiquitinating enzyme subfamily as substrates of the von Hippel–Lindau tumor suppressor.* Biochemical and Biophysical Research Communications, 2002. **294**(3): p. 700-709.

22. Na, X., et al., *Identification of the RNA polymerase II subunit hsRPB7 as a novel target of the von Hippel—Lindau protein*. Vol. 22. 2003. 4249-4259.

23. Yang, H., et al., *pVHL Acts as an Adaptor to Promote the Inhibitory Phosphorylation of the NF-κB Agonist Card9 by CK2.* Molecular Cell, 2007. **28**(1): p. 15-27.

24. Hergovich, A., et al., *Regulation of microtubule stability by the von Hippel-Lindau tumour suppressor protein pVHL.* Nat Cell Biol, 2003. **5**(1): p. 64-70.

25. Lolkema, M.P., et al., *The von Hippel–Lindau tumour suppressor interacts with microtubules through kinesin-2.* FEBS Letters, 2007. **581**(24): p. 4571-4576.

26. Lolkema, M.P., et al., *The von Hippel–Lindau tumor suppressor protein influences microtubule dynamics at the cell periphery.* Experimental Cell Research, 2004. **301**(2): p. 139-146.

27. Pal, S., et al., *Activation of Sp1-mediated Vascular Permeability Factor/Vascular Endothelial Growth Factor Transcription Requires Specific Interaction with Protein Kinase C ζ.* Journal of Biological Chemistry, 1998. **273**(41): p. 26277-26280.

28. Zhou, M.I., et al., *Tumor Suppressor von Hippel-Lindau (VHL) Stabilization of Jade-1 Protein Occurs through Plant Homeodomains and Is VHL Mutation Dependent.* Cancer Research, 2004. **64**(4): p. 1278-1286.

29. Zhou, M.I., et al., *The von Hippel-Lindau Tumor Suppressor Stabilizes Novel Plant Homeodomain Protein Jade-1.* Journal of Biological Chemistry, 2002. **277**(42): p. 39887-39898.

30. Berndt, J.D., R.T. Moon, and M.B. Major, *β-catenin gets jaded and von Hippel-Lindau is to blame.* Trends in Biochemical Sciences, 2009. **34**(3): p. 101-104.

31. Datta, K., et al., *Inhibition of Insulin-like Growth Factor-I-mediated Cell Signaling by the von Hippel-Lindau Gene Product in Renal Cancer.* Journal of Biological Chemistry, 2000. **275**(27): p. 20700-20706.

32. Roe, J.S., et al., *p53 stabilization and transactivation by a von Hippel-Lindau protein.* Mol Cell, 2006. **22**(3): p. 395-405.

33. Roe, J.S., et al., *Phosphorylation of von Hippel-Lindau protein by checkpoint kinase 2 regulates p53 transactivation.* Cell Cycle, 2011. **10**(22): p. 3920-8.

34. Roe, J.S. and H.D. Youn, *The positive regulation of p53 by the tumor suppressor VHL.* Cell Cycle, 2006. **5**(18): p. 2054-6.

35. Semenza, G.L., *VHL and p53: tumor suppressors team up to prevent cancer.* Mol Cell, 2006. **22**(4): p. 437-9.

36. Tyers, M. and R. Rottapel, *VHL: A very hip ligase.* Proceedings of the National Academy of Sciences, 1999. **96**(22): p. 12230-12232.

37. Danilin, S., et al., *Role of the RNA-binding protein HuR in human renal cell carcinoma.* Carcinogenesis, 2010. **31**(6): p. 1018-1026.

38. Datta, K., et al., *Role of elongin-binding domain of von hippel lindau gene product on HuR-mediated VPF//VEGF mRNA stability in renal cell carcinoma.* Oncogene, 2005. **24**(53): p. 7850-7858.

39. Yuen, J.S.P., et al., *The VHL tumor suppressor inhibits expression of the IGF1R and its loss induces IGF1R upregulation in human clear cell renal carcinoma.* Oncogene, 2007. **26**(45): p. 6499-6508.

40. Lolkema, M.P., et al., *Tumor Suppression by the von Hippel-Lindau Protein Requires Phosphorylation of the Acidic Domain.* Journal of Biological Chemistry, 2005. **280**(23): p. 22205-22211.

41. Corn, P.G., et al., *Tat-binding protein-1, a component of the 26S proteasome, contributes to the E3 ubiquitin ligase function of the von Hippel-Lindau protein.* Nat Genet, 2003. **35**(3): p. 229-237.

42. Mans, D.A., et al., *Mobility of the von Hippel-Lindau tumour suppressor protein is regulated by kinesin-2.* Exp Cell Res, 2008. **314**(6): p. 1229-36.

43. Li, Z., et al., *The VHL protein recruits a novel KRAB‐A domain protein to repress HIF‐1α transcriptional activity*. Vol. 22. 2003. 1857-1867.
